# Psychosocial interventions to reduce alcohol consumption in concurrent problem alcohol and illicit drug users: Cochrane Review^a^

**DOI:** 10.1186/2046-4053-2-3

**Published:** 2013-01-12

**Authors:** Jan Klimas, Catherine-Anne Field, Walter Cullen, Clodagh SM O’Gorman, Liam G Glynn, Eamon Keenan, Jean Saunders, Gerard Bury, Colum Dunne

**Affiliations:** 1 Graduate Entry Medical School, Faculty of Education and Health Sciences, University of Limerick, Limerick, Ireland; 2 School of Medicine and Medical Science, University College Dublin, Coombe Healthcare Centre, Dolphins Barn Street, Dublin 8, Ireland; 3 Centre for Interventions in Infection, Inflammation & Immunity (4i), Faculty of Education and Health Sciences, University of Limerick, Limerick, Ireland; 4 Department of Paediatrics, Mid-Western Regional Hospital, Limerick, Ireland; 5 Department of General Practice, National University of Ireland, Galway, Ireland; 6 Addiction Services, Health Service Executive, Dublin, Ireland; 7 Statistical Consulting Unit/ Applied Biostatistics Consulting Centre /CSTAR, Graduate Entry Medical School, University of Limerick, Limerick, Ireland

**Keywords:** Alcohol, Brief intervention, Illicit drugs, Opioids, Systematic review, Screening, Methadone

## Abstract

**Background:**

Problem alcohol use is common among illicit drug users and is associated with adverse health outcomes. It is also an important factor in poor prognosis among drug users with hepatitis C virus (HCV) as it impacts progression to hepatic cirrhosis or opiate overdose in opioid users. The aim of this systematic review was to assess the effects of psychosocial interventions for problem alcohol use in adult illicit drug users with concurrent problem alcohol use (principally, problem drug users of opiates and stimulants).

**Methods:**

We searched the following databases (November 2011): Cochrane Library, PUBMED, EMBASE, CINAHL, PsycINFO and reference list of articles. We also searched conference proceedings and online registers of clinical trials. Two reviewers independently assessed risk of bias and extracted data from included randomized controlled trials.

**Results:**

Four studies (594 participants) were included in this review. Half of the trials were rated as having a high or unclear risk of bias. The four studies considered six different psychosocial interventions grouped into four comparisons: 1) cognitive-behavioral coping skills training versus 12-step facilitation (N = 41), 2) brief intervention versus treatment as usual (N = 110), 3) hepatitis health promotion versus motivational interviewing (N = 256), and 4) brief motivational intervention versus assessment-only group (N = 187). Differences between studies precluded any pooling of data. Findings are described for each trial individually. Most findings were not statistically significant except for comparison 2: decreased alcohol use at three months (risk ratio (RR) 0.32; 95% confidence interval (CI) 0.19 to 0.54) and nine months (RR 0.16; 95% CI 0.08 to 0.33) in the treatment-as-usual group and comparison 4: reduced alcohol use in the brief motivational intervention (RR 1.67; 95% CI 1.08 to 2.60).

**Conclusions:**

No conclusion can be made because of the paucity of the data and the low quality of the retrieved studies.

## Background

Problem alcohol use is common among illicit drug users and is associated with adverse health outcomes, which include physical, psychological and social implications [1-4]. Recent NIDA (National Institute on Drug Abuse) meta-analyses of US clinical trials found alcohol use disorders (AUDs) in 38% and 45% of opiate- and stimulant-using treatment seekers, respectively [5,6].

Problem drug users are at high risk of liver disease resulting from hepatitis C virus (HCV) infection because of its high prevalence in this population [7]. Problem alcohol use is an important factor in determining poor prognosis among people with HCV as it impacts progression to hepatic cirrhosis, increased HCV-ribonucleic acid (RNA) levels or fatal opiate overdose in opiate users [8,9].

Psychosocial interventions are best described as ‘psychologically-based interventions aimed at reducing consumption behavior or alcohol-related problems’ [10], which exclude any pharmacological treatments. The most frequently used interventions include motivational interviewing (MI), cognitive-behavioral therapy (CBT), psychodynamic approaches, screening and brief interventions (SBI), family therapy, drug counseling, 12-step programs, therapeutic communities (TC) and vocational rehabilitation (VR). For descriptions, see the review by Amato *et al*. [11].

Substantial evidence has described the value of psychosocial interventions in treating problem alcohol use [12-15]. Even in their brief version, psychosocial interventions are feasible and potentially highly effective components of an overall public health approach to reducing problem alcohol use, although considerable variation in effectiveness trials exists and problem drug users from non-specialist settings (for example, primary care) are under-represented in these trials [10,16].

Two previous narrative reviews of literature have dealt with the question being asked in this review, to date. The older of these reviews discussed six reports of four studies among methadone patients and saw some promise in the contingency management procedures [17]. A more recent review described implications of combining behavioral and pharmacological treatments that are effective in treating either alcohol- or drug-use disorders alone, for the treatment of people who have both of these disorders [18]. While pointing to the paucity of research specifically focused on the treatment of people with co-occurring alcohol and other substance use disorders, the reviews concluded that successful treatment must take into account both alcohol- and drug-use disorders.

The lack of systematic evaluation, together with the anticipated differences in the responsiveness of problem drug users to psychosocial interventions, provides additional reasons for conducting this review [19].

## Objective

This article provides a comprehensive summary of the 62-page systematic review assessing interventions for problem alcohol use in illicit drug users, published in the Cochrane Library [20]. The aim of the systematic review was to determine the effectiveness of psychosocial interventions targeting problem alcohol use versus other treatments in illicit drug users, especially the effectiveness of these interventions on reducing alcohol consumption.

## Methods

### Searching and study selection

Only studies that defined participants as adult (≥ 18 years) problem drug and alcohol users at randomization were included. Problem drug use was defined by the European Monitoring Centre for Drugs and Drug Addiction as ‘injecting drug use or long-duration/regular use of opioids, cocaine and/or amphetamines’ [21]. The considered interventions were any psychosocial interventions described by the study’s author as such (for example, motivational interviewing, brief intervention, cognitive behavioral therapy, contingency management, family therapy, *etcetera*).

The outcomes assessed were 1) alcohol use (reduction or stabilization) as measured by either biological markers or self-report tests; 2) illicit drug use (changes in illicit drug use) as measured by either biological markers or self-report tests; 3) engagement in further treatment (that is, drop-out rates, utilization of health services); 4) alcohol-related problems or harms as represented by physical or mental health outcomes associated with problem alcohol use. We planned to pool the results from individual trials if a sufficient number of studies used a measure of alcohol problems and the included studies utilized similar instruments to measure their outcomes.

An all-language search (November 2011) identified trials in MEDLINE (since 1966), CINAHL (since 1982), *The Cochrane Library*, (Issue 11, Nov 2011), PsycINFO (since 1872), and EMBASE (since 1974). Databases were searched using a strategy developed incorporating the filter for the identification of RCTs [22], combined with selected MeSH terms and free-text terms relating to alcohol use (See search strategy in Table S6, Additional file 1).

We also searched reference lists of articles considered eligible based on full report screening and other relevant papers; conference proceedings; controlled trial registers; and contacted investigators and relevant trial authors seeking information about unpublished or incomplete trials.

Included studies were randomized controlled trials (RCTs) or clinical trials (CCTs) that compared psychosocial intervention to other psychosocial interventions, standard care, no intervention, waiting list, placebo/or any other non-pharmacological therapy (including moderate drinking, assessment only). Multiple-arm trials were included if they had at least two psychosocial arms.

### Data extraction and analysis

Two authors screened lists of citations and abstracts independently. Differences between selection lists were resolved by discussion with two other review authors with respective thematic and methodological expertise. Full texts of all potentially relevant records were retrieved and data were extracted independently by two authors from the full-text reports, using an electronic version of an amended data extraction form of the Cochrane Drug and Alcohol review group (CDAG).

Quality assessments were performed independently by two authors using the criteria recommended by the *Cochrane Handbook for Systematic Reviews of Interventions*[22]: the domains of sequence generation, allocation concealment blinding of outcome assessor (separately for objective and subjective outcomes) and incomplete outcome data (end of the study and results at follow-up).

A formal meta-analysis was not possible owing to substantial differences between studies; there were no two studies similar enough to be considered for pooling. Results of included studies are reported individually for each trial, re-expressed as RRs for dichotomous outcomes and MDs for continuous outcomes, and reported with 95% Confidence Intervals (CIs). A fixed-effect model was used because there was only one study for each comparison.

### Ethical considerations

This systematic review adheres to the Preferred Reporting Items for Systematic Reviews and Meta-Analyses (PRISMA) guidelines [23].

## Results

### Study flow, trial characteristics, and quality assessment

**Figure 1:**
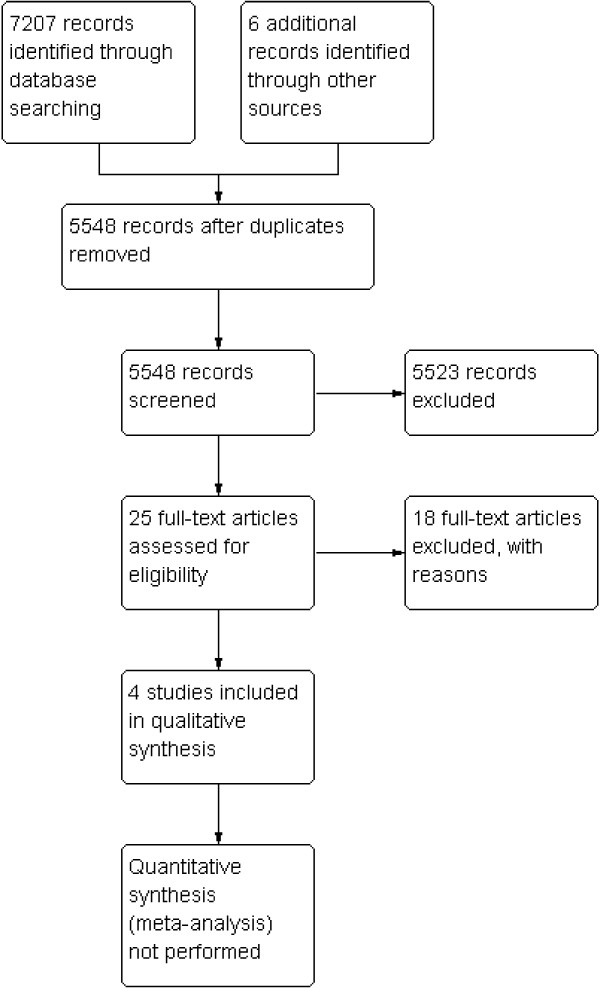
Flow chart for studies in the systematic review.

The process and results of study identification are outlined in a flow diagram (Figure 1) according to the PRISMA statement [23].

Four studies (594 participants) were eligible for this review. The studies assessed the effectiveness of six psychosocial interventions: CBT, 12-step facilitation (TSF), BI, hepatitis health promotion (HHP), MI, and brief motivational intervention (BMI).

The types of psychosocial intervention and setting are as follows:

1. CBT versus TSF in an outpatient clinic [24].

2. BI versus treatment as usual in an outpatient clinic with/without opioid substitution treatment [25].

3. MI (group) versus HHP in an opioid substitution clinic [26].

4. MI (single) versus HHP in an opioid substitution clinic [26].

5. BMI versus assessment only in a needle exchange program [27].

Three studies were conducted in USA and one study was conducted in Switzerland. Trial duration ranged from 4 to 12 weeks (plus various follow-ups) with a mean duration of 7.5 weeks. Between one and 16 sessions were offered to participants, with a mean of 5.5 sessions being offered (from 15 minutes to 16 hours of treatment time).

Of the 594 participants, all were problem drug users^b^. Thirty-three percent of the participants were female. Mean age was 38.3 years.

See Characteristics of included studies table in Table S7, Additional file 2 for more detailed information. Methodological quality of the included studies was generally considered as low. Random sequence generation was judged as adequate in two studies, while only one study was judged being at a low risk of bias, one was judged at a high risk of bias and the remaining at an unclear risk of bias. Participants and personnel were not blinded in all studies to the kind of interventions involved, and objective outcomes were not reported in the trials. They were used as an additional measure to confirm abstinence in two studies.

For subjective outcomes, participants and personnel were not blinded in all studies to the kind of interventions; two studies (50%) specified that outcome assessors were blinded and were judged to be at low risk of bias. Two studies reported that the outcome assessor was not blinded and were judged at a high risk of bias; for one of these this is unpublished information from email communication with the study authors. For incomplete outcome data, three studies were judged to be at low risk of bias because only a few patients (less than 10%) withdrew from the studies, or inversely there was a high rate of drop-out, but percentages were balanced across intervention groups and reasons for withdrawing were provided, or authors performed an intention to treat (ITT) analysis. One study was judged to be at a high risk of bias because of a high drop-out rate, which was unbalanced across groups.

### Effects of interventions

Meta-analysis of all included studies was not possible (complete data available from the first author). The results were summarized according to the type of psychosocial intervention, with comparisons of quantitative data where possible. The included studies used different questionnaires to measure their outcomes and for many of them the authors did not report the post-treatment/follow-up scores or they did not state what was considered to represent mild, moderate and severe categories. This prevented comparison of results across studies. One study had three arms; in this case they were entered into two separate comparisons (group and single format), so they were not counted twice. Tables 1, 2, 3, 4, 5 below present the effects of the interventions by comparisons examined in the primary studies. Primary outcome was alcohol use or abstinence and secondary outcome was illicit drug use or abstinence.

**Table 1:** **Cognitive**-**behavioral coping skills training** (**CBT**) **versus 12**-**step facilitation** (**TSF**)

**Outcome or subgroup**	**Studies**	**Participants**	**Statistical method**	**Effect estimate**
*1*.*1 Continuous outcomes*			*Mean Difference* (*IV*, *Fixed*, *95*% *CI*)	*Subtotals only*
1.1.1 Alcohol abstinence as maximum number of weeks of consecutive alcohol abstinence during treatment	1	41	Mean Difference (IV, Fixed, 95% CI)	0.40 [-1.14, 1.94]
1.1.2 Illicit drug abstinence as maximum number of weeks of consecutive abstinence from cocaine during treatment	1	41	Mean Difference (IV, Fixed, 95% CI)	0.80 [-0.70, 2.30]
*1*.*2 Dichotomous outcomes*			*Risk Ratio* (*M*-*H*, *Fixed*, *95*% *CI*)	*Subtotals only*
1.2.1 Alcohol abstinence as number achieving three or more weeks of consecutive alcohol abstinence during treatment	1	41	Risk Ratio (M-H, Fixed, 95% CI)	1.96 [0.43, 8.94]
1.2.2 Illicit drug abstinence as number achieving three or more weeks of consecutive abstinence from cocaine during treatment	1	41	Risk Ratio (M-H, Fixed, 95% CI)	1.10 [0.42, 2.88]
1.2.3 Alcohol abstinence during follow-up year	1	41	Risk Ratio (M-H, Fixed, 95% CI)	2.38 [0.10, 55.06]
1.2.4 Illicit drug abstinence as abstinence from cocaine during follow-up year	1	41	Risk Ratio (M-H, Fixed, 95% CI)	0.39 [0.04, 3.98]

**Table 2:** **Brief intervention** (**BI**) **versus treatment as usual**

**Outcome or subgroup**	**Studies**	**Participants**	**Statistical method**	**Effect estimate**
*2*.*1 Continuous outcomes*			*Mean Difference* (*IV*, *Fixed*, *95*% *CI*)	*Subtotals only*
2.1.1 Alcohol use as AUDIT scores at three months	1	110	Mean Difference (IV, Fixed, 95% CI)	0.10 [-2.96, 3.16]
2.1.2 Alcohol use as AUDIT Scores at nine months	1	110	Mean Difference (IV, Fixed, 95% CI)	1.50 [-1.74, 4.74]
2.1.3 Alcohol use as number of drinks per week at three months	1	110	Mean Difference (IV, Fixed, 95% CI)	2.40 [-4.59, 9.39]
2.1.4 Alcohol use as number of drinks per week at nine months	1	110	Mean Difference (IV, Fixed, 95% CI)	−1.70 [-8.93, 5.53]
*2*.*2 Dichotomous outcomes*			*Risk Ratio* (*M*-*H*, *Fixed*, *95*% *CI*)	*Subtotals only*
2.2.1 Alcohol use as decreased alcohol use at three months	1	110	Risk Ratio (M-H, Fixed, 95% CI)	0.32^a^ [0.19, 0.54]
2.2.2 Alcohol use as decreased alcohol use at nine months	1	110	Risk Ratio (M-H, Fixed, 95% CI)	0.16^a^ [0.08, 0.33]

^*a*^significance *P* <0.0001.

**Table 3:** **Motivational interviewing** (**group**) (**MI**-**G**) **versus hepatitis health promotion** (**HHP**)

**Outcome or subgroup**	**Studies**	**Participants**	**Statistical method**	**Effect estimate**
*3*.*1 Continuous outcomes*			*Mean Difference* (*IV*, *Fixed*, *95*% *CI*)	*Subtotals only*
3.1.1 Alcohol use as number of standard drinks consumed per day over the last 30 days	1	147	Mean Difference (IV, Fixed, 95% CI)	−0.40 [-2.03, 1.23]
3.1.2 Illicit drug use as frequency of drug use (as measured by Addiction Severity Index - ASI drug)	1	147	Mean Difference (IV, Fixed, 95% CI)	0.00 [-0.03, 0.03]
3.1.3 Illicit drug use as a composite drug score (frequency*severity for all drugs taken)	1	151	Mean Difference (IV, Fixed, 95% CI)	0.0[-0.42, 0.42]
*3*.*2 Dichotomous outcomes*			*Risk Ratio* (*M*-*H*, *Fixed*, *95*% *CI*)	*Subtotals only*
3.2.1 Alcohol use as greater than 50% reduction in number of standard drinks consumed per day over the last 30 days	1	166	Risk Ratio (M-H, Fixed, 95% CI)	1.10 [0.82, 1.48]
3.2.2 Alcohol abstinence as abstinence from alcohol over the last 30 days	1	166	Risk Ratio (M-H, Fixed, 95% CI)	0.88 [0.49, 1.58]

**Table 4:** **Motivational interviewing** (**single**) (**MI**-**S**) **versus hepatitis health promotion** (**HHP**)

**Outcome or subgroup**	**Studies**	**Participants**	**Statistical method**	**Effect estimate**
*4*.*1 Continuous outcomes*			*Mean Difference* (*IV*, *Fixed*, *95*% *CI*)	*Subtotals only*
4.1.1 Alcohol use as number of standard drinks consumed per day over the last 30 days	1	155	Mean Difference (IV, Fixed, 95% CI)	−0.10 [-1.89, 1.69]
4.1.2 Illicit drug use as frequency of drug use (as measured by Addiction Severity Index - ASI drug)	1	155	Mean Difference (IV, Fixed, 95% CI)	0.00 [-0.03, 0.03]
4.1.3 Illicit drug use as a composite drug score (frequency/severity for all drugs taken)	1	157	Mean Difference(IV, Fixed, 95% CI)	−0.10 [-0.46, 0.26]
*4*.*2 Dichotomous outcomes*			*Risk Ratio* (*M*-*H*, *Fixed*, *95*% *CI*)	*Subtotals only*
4.2.1 Alcohol use as greater than 50% reduction in number of standard drinks consumed per day over the last 30 days	1	177	Risk Ratio (M-H, Fixed, 95% CI)	0.92 [0.68, 1.26]
4.2.2 Alcohol abstinence as abstinence from alcohol over the last 30 days	1	177	Risk Ratio (M-H, Fixed, 95% CI)	0.97 [0.56, 1.67]

**Table 5:** **Brief motivational intervention** (**BMI**) **versus assessment only**

**Outcome or subgroup**	**Studies**	**Participants**	**Statistical method**	**Effect estimate**
*5*.*1 Continuous outcomes*			*Mean Difference* (*IV*, *Fixed*, *95*% *CI*)	*Subtotals only*
5.1.1 Alcohol use as number of days in the past 30 days with alcohol use at one month	1	187	Mean Difference (IV, Fixed, 95% CI)	−0.30 [-3.38, 2.78]
5.1.2 Alcohol use as number of days in the past 30 days with alcohol use at six months	1	187	Mean Difference (IV, Fixed, 95% CI)	−1.50 [-4.56, 1.56]
*5*.*2 Dichotomous outcomes*			*Risk Ratio* (*M*-*H*, *Fixed*, *95*% *CI)*	*Subtotals only*
5.2.1 Alcohol use as 25% reduction of drinking days in the past 30 days	1	187	Risk Ratio (M-H, Fixed, 95% CI)	1.23 [0.96, 1.57]
5.2.2 Alcohol use as 50% reduction of drinking days in the past 30 days	1	187	Risk Ratio (M-H, Fixed, 95% CI)	1.27 [0.96, 1.68]
5.2.3 Alcohol use as 75% reduction of drinking days in the past 30 days	1	187	Risk Ratio (M-H, Fixed, 95% CI)	1.21 [0.84, 1.75]
5.2.4 Alcohol use as one or more drinking days’ reduction in the past 30 days	1	187	Risk Ratio (M-H, Fixed, 95% CI)	1.12 [0.91, 1.38]
5.2.5 Alcohol use as seven or more drinking days’ reduction in the past 30 days	1	187	Risk Ratio (M-H, Fixed, 95% CI)	1.67^a^ [1.08, 2.60]

^a^Significance *P* = 0.02.

Most of the comparisons were not statistically significant, except for decreased alcohol use at three months (RR 0.32; 95% CI 0.19 to 0.54) and nine months (RR 0.16; 95% CI 0.08 to 0.35) in the study by Feldman *et al*. [25]. These results favored the control intervention. Also, participants receiving BMI were significantly more likely to reduce their alcohol use by seven or more days in the past 30 days at six months, compared to control group (RR 1.67; 95% CI 1.08 to 2.60) [27].

## Discussion

Four studies involving 594 participants were included in this review. The studies assessed the effectiveness of six psychosocial interventions: CBT, TSF, BI, HHP, MI, and BMI. There was significant clinical and methodological heterogeneity among the included studies, which precluded meta-analysis. Comparing different psychosocial interventions, there was only one study for each comparison. Most of the comparisons were not statistically significant, except for decreased alcohol use at three months and nine months in the BI study [25]. Surprisingly, these results favored the control intervention. This could be interpreted in light of the main limitations of this study, namely, the standard intervention provided to the control group was ‘too strong’ to enable reasonable comparison with the intervention group, and the intervention group had a high proportion of people with alcohol addiction who received the 15-minute-long brief alcohol intervention. This is in contradiction to the manual for BIs, which states that people with alcohol addiction should not receive BI, but should be referred to a specialized, more intensive treatment [28]. Also other systematic reviews examining the general population indicated that BI was effective for harmful/ hazardous use, but not for dependence [12,15]. Finally, participants receiving BMI were significantly more likely to reduce their alcohol use by seven or more days in the past 30 days at six months’ follow-up, compared to the control group [27].

Our review was systematic, but not without weaknesses. We did not limit our searches to studies published in English; however, studies in non-English languages may have been missed because they are commonly less frequently indexed in the selected databases. Unpublished studies may also have been missed. Unpublished studies are likely to have negative results, which is why they are not published. The major limitation of the review process was that most trials did not provide enough published data or did not provide data in a form that could be extracted for meta-analysis. Although the lead authors of all four studies were emailed, only two responded and provided further data. Furthermore, we could not include a number of potentially relevant studies, because they involved drug users without problem alcohol use in their samples.

Similar to our work, two previous narrative reviews were unable to identify evidence to answer our question or to conduct a meta-analysis [17,18]. Subsequently, they based their conclusions on evidence coming from mixed-type studies (for example, case studies and RCTs) or studies that included illicit drug users without a concurrent problem alcohol use. We excluded these types of studies. Furthermore, the review by Arias *et al*. [18] discussed 14 reports/studies related to treatment of co-occurring alcohol and cocaine/opioid dependence, two of which were included in our review.

This review is unintentionally tapping into an important question: what constitutes standard maintenance/outpatient treatment? It appears that all standard treatments contain some type of psychosocial support, which varies considerably, and this makes it difficult to evaluate the added value of additional services. This was true for studies included in our review and, in addition, the process of assessment or quick feedback following the assessment, or both, resulted in improved alcohol outcomes among the participants.

## Conclusions

Based on the weak evidence identified in this review, we cannot recommend using or ceasing psychosocial interventions for problem alcohol use in illicit drug users. Similar to other conditions, problem alcohol use has better prospects for a successful treatment if approached early. Evidence from the general population suggests that we need to focus on early *detection and* intervention as well as try to influence more established alcohol patterns of use. Early interventions are not implemented into routine care, especially in the settings where there is a potential for impact owing to high exposure, such as primary care [29-32]. Given the high rates of co-occurrence of alcohol and drug problems, integration of alcohol- and drug- orientated interventions appears as a logical action, but in light of this review remains without an evidence base.

This review emphasizes the need for randomized controlled trials (RCT) to test the effectiveness of psychosocial interventions in reducing problem alcohol use in illicit drug users. We recommend RCTs of robust methodology that are well reported to allow for critical appraisal.

## Endnote

^a^This article is an abridged version of a Cochrane Review recently published in the Cochrane Database of Systematic Review 2012, Issue 11, http://dx.doi.org/10.1002/14651858.CD009269.pub2 (See http://www.thecochranelibrary.com for information). Cochrane reviews are regularly updated as new evidence emerges and in response to feedback. Consult Cochrane Database of Systematic Reviews for the most recent version of the review.

^b^One multi-arm trial included 122 participants [24]; however, only two psychosocial arms (N = 41) were considered for this review.

## Abbreviations

AUDIT: Alcohol Use Disorders Identification Test; AUDs: Alcohol use disorders; BI: Brief interventions; BMI: Brief motivational intervention; CBT: Cognitive-behavioral therapy; CCTs: Controlled clinical trials; CDAG: Cochrane Systematic Reviews and Meta-analyses; CI: Confidence interval; EPICOT: Evidence, population, intervention, comparison, outcomes, time stamp; HCV: Hepatitis C virus; HHP: Hepatitis health promotion; MI: Motivational interviewing; MMT: Methadone maintenance treatment; NIDA: National Institute on Drug Abuse; NIH: National Institutes of Health; RCTs: Randomized controlled trials; RNA: HCV-ribonucleic acid; RR: Risk ratio; SBI: Screening and brief interventions; TC: Therapeutic communities; TSF: 12-step facilitation; VR: Vocational rehabilitation.

## Competing interests

All authors declared that they have no competing interest.

## Authors’ contributions

JK designed and coordinated the review and wrote and re-drafted the protocol and full review. WC, CAF, and COG contributed to the design of the review and commented on drafts. LG and JS: provided methodological advice and commented on review drafts. GB, EK, and CD commented on review drafts. All authors read and approved the final manuscript.

## Supplementary Material

Additional file 1: Table S6 Medline search strategy. Click here for file

Additional file 2: Table S7 Characteristics of included randomiszed controlled trials. Click here for file
