# Supplementary material for: Psychosocial interventions to reduce alcohol consumption in concurrent problem alcohol and illicit drug users: Cochrane Reviewa
Source: Syst Rev. 2013 Jan 12;2:3. doi: 10.1186/2046-4053-2-3 (PMC3564788; doi:10.1186/2046-4053-2-3)
Supplement: Additional file 1: Table S6 — Medline search strategy. [file 2046-4053-2-3-S1.docx]

**Table S6** **Medline search strategy***

| No | **Database: PubMed** | Results |
| --- | --- | --- |
|  | **Date: 22 November 2011** |  |
| #34 | Search **(((#3) AND #9) AND #15) AND #33** | 3726 |
| #33 | Search **((((((((((((((((#16) OR #17) OR #18) OR #19) OR #20) OR #21) OR #22) OR #23) OR #24) OR #25) OR #26) OR #27) OR #28) OR #29) OR #30) OR #31) OR #32** | 1187926 |
| #32 | Search **“case management”[Mesh]** | 6991 |
| #31 | Search **“Relaxation Therapy”[Mesh]** | 6087 |
| #30 | Search **“social support”[Mesh]** | 39581 |
| #29 | Search **“self-control training”[tiab]** | 37 |
| #28 | Search **“coping skill”[tiab]** | 54 |
| #27 | Search **neurobehavioral*[tiab]** | 5439 |
| #26 | Search **“supportive expressive therapy” [tiab]** | 14 |
| #25 | Search **“stress management training” [tiab]** | 150 |
| #24 | Search **“social skill”[tiab]** | 262 |
| #23 | Search **“family therapy” [tiab]** | 2272 |
| #22 | Search **“cognitive therapy” [tiab]** | 1344 |
| #21 | Search **“counseling"[Mesh] or counsel*[TIAB]** | 69913 |
| #20 | Search **“minimal intervention” [tiab]** | 450 |
| #19 | Search **“early intervention”[tiab]** | 6813 |
| #18 | Search **“brief intervention”[tiab]** | 923 |
| #17 | Search **incentive*[tiab] OR voucher[tiab] or psychotherap*[tiab] or psychosocial*[tiab] or “behavior therapy” [tiab] OR “behavior therapy”[tiab] or reinforcement[tiab] or motivation*[tiab] or contingent*[tiab] or advice[tiab] or biofeedback[tiab] or community[tiab] or stimulation[tiab] or education*[tiab]** | 1010905 |
| #16 | Search **psychotherapy [Mesh]** | 129903 |
| #15 | Search **((((#10) OR #11) OR #12) OR #13) OR #14** | 457865 |
| #14 | Search **“Drinking behavior”[mesh]** | 46406 |
| #13 | Search **alcoholic Intoxication [mesh]** | 9764 |
| #12 | Search **alcoholism[mesh]** | 61049 |
| #11 | Search **consumption[tiab] or binge[tiab] or intoxication[tiab] or abuse[tiab] or misuse[tiab] or drink*[tiab]** | 305069 |
| #10 | Search **alcohol*[tiab]** | 192874 |
| #9 | Search **((((#4) OR #5) OR #6) OR #7) OR #8** | 352933 |
| #8 | Search **“Designer Drugs”[Mesh]** | 520 |
| #7 | Search **“Street Drugs”[Mesh]** | 7119 |
| #6 | Search **drug[tiab] or polydrug[tiab] or substance[tiab] or opioid[tw] or opiate[tw] or hallucinogen[tiab] or cocaine [tw] benzodiazepine*[tw] or amphetamine*[tw] or “anti-anxiety-agents”[tiab] or barbiturate*[tiab] or “lysergic acid”[tiab] or ketamine[tiab] or cannabis[tiab] or marihuana[tiab] or hashish[tiab] or opium[tiab] or inhalant*[tiab] or solvent[tiab] or steroid*[tiab] or methadone[tiab] or morphine[tiab] or ecstasy[tiab] OR MDMA[tiab]** | 333122 |
| #5 | Search **narcotic*[tiab]** | 11504 |
| #4 | Search **“heroin"[Mesh] OR heroin[tiab]** | 10705 |
| #3 | Search **(#1) OR #2** | 2519843 |
| #2 | Search **addict*[tiab] or overdose[tiab] or intoxicat*[tiab] or abstin*[tiab] or abstain*[tiab] or withdrawal*[tiab] or abuse*[tiab] or use*[tiab] or misuse[tiab] or disorder*[tiab] or dependen*[tiab]** | 2323846 |
| #1 | Search **“substance related disorders”[Mesh]** | 317590 |

** Search slightly amended for other databases.*
