# Supplementary material for: Psychosocial interventions to reduce alcohol consumption in concurrent problem alcohol and illicit drug users: Cochrane Reviewa
Source: Syst Rev. 2013 Jan 12;2:3. doi: 10.1186/2046-4053-2-3 (PMC3564788; doi:10.1186/2046-4053-2-3)
Supplement: Additional file 2: Table S7 — Characteristics of included randomiszed controlled trials. [file 2046-4053-2-3-S2.docx]

**Additional file 2**

**Table S7** **Characteristics of included randomized controlled trials**

| ***Item/ Study*** | **Carroll et al. 1998** | **Feldman et al. 2011** | **Nyamathi et al. 2010** | **Stein et al. 2002** |
| --- | --- | --- | --- | --- |
| **Methods** | Study design: RCT, single blind | Study design: RCT | Study design: RCT open label, 3 arms | Study design: RCT |
|  |  | Recruitment modality of participants: for 1 year, participation in the study was proposed systematically to each adult outpatient who was treated for opioid or cocaine dependence |  | Recruitment modality of participants: study was advertised at 3 NEP sites using posters and NEP volunteers offered all clients referral cards. NEP clients called a study telephone to be screened by a research assistant at a separate research site in hospital. |
|  |  |  | Recruitment modality of participants: flyers displayed in 5 methadone treatment sites |  |
|  | Recruitment modality of participants: individuals seeking treatment at the outpatient treatment unit of the APT Foundation, or from respondents to newspaper advertisements or public service announcements |  |  |  |
| **Participants** | N = 122 (41 in 2 arms selected for this review) | N = 110 | N = 256 | N = 187 |
|  |  | Gender: 72.3% male | Gender: 59.2% male | Gender: 63.6% male |
|  |  | Age (mean ± SD): 35 ± 7.8 years | Age (mean ± SD): 51.2 ± 8.4 years | Age: mean 36.2 years |
|  |  |  |  | Condition: problem alcohol use, that is, AUDIT-positive (> 8) active IDUs. “Current alcohol abuse or dependence diagnosis was ascertained using the SCID interview. 159 (85.0%) met DSM-IV criteria for current alcohol abuse or dependence (80% for abuse, 70% for dependence).” |
|  | Gender: 27% female | Condition: problem alcohol use based on questions from the AUDIT questionnaire, that is, excessive drinking (7 ≤ AUDIT score < 13 for men and 6 ≤ AUDIT score < 13 for women); and alcohol dependence (score > 13); 43.8% were classified as excessive drinkers and 56.2% as alcohol dependents. | Condition: reported moderate-to-heavy alcohol use based on questions from the ASI. Methadone maintenance treatment was an inclusion criterion (minimum 3 months) |  |
|  | Age (mean ± SD): 30.8 ± 5.5 years |  |  |  |
|  | Condition: “All subjects met current DSM-III-R criteria for cocaine dependence, and for concurrent alcohol dependence (85%) or alcohol abuse (15%)” |  |  |  |
| **Interventions** | Description of the experimental and control interventions: | Description of the experimental and control interventions: the intervention group was BI and the control group was TAU. | Description of the experimental and control interventions: (1) nurse-led HHP group sessions; (2) MI delivered in group sessions (MI-group), and (3) MI delivered 1-on-1 sessions (MI-single). | Description of the experimental and control interventions: (1) brief MI and (2) control group |
|  | The trial included 5 treatment arms: CBT plus disulphiram; TSF plus disulphiram; CM plus disulphiram; CBT plus no medication; TSF plus no medication. We considered only the latter 2 psychosocial arms. CBT was based on Marlatt’s relapse prevention model and TSF was adapted from that used in Project MATCH and was grounded in the concept of substance dependence as a spiritual and medical disease |  |  | (1) MI: focus on alcohol use and HIV risk-taking |
|  |  |  |  | Goals: to assess the degree to which the patient engages in hazardous drinking; to identify relationships between alcohol consumption and alcohol-related negative consequences including HIV risk behavior; to identify goals for behavior change and any barriers to change |
|  |  | (1) BI: BI was delivered in 1 session, based on WHO guidelines, delivered by a trained staff (4 hours’ training). The intervention group received the same TAU as controls. The outpatient staff consisted of a psychiatrist, general practitioner, psychologist, nurse, and social worker |  |  |
|  |  |  | (1) HHP: manualized, didactic style, also interactive as the group raised questions. Delivered by a nurse and hepatitis-trained research assistant. Sessions based on “The comprehensive health seeking and coping paradigm (CHSCP; Nyamathi, 1989)”. Focus: progression of HCV infection and the culturally-sensitive strategies that infected individuals can adopt to prevent or reduce accumulated damage to liver functioning. |  |
|  |  |  |  | • Included a written change plan, designed to reduce the link between alcohol consumption and hazardous behaviours that may lead to negative consequences of drinking, including HIV risk behaviour |
|  |  | (2) TAU: “The control group received TAU in addition to AUDIT and score feedback. TAU refers to outpatient pharmacological and psychosocial treatment. Maintenance treatment with methadone or heroin included medical and psychiatric follow-up, primary health care, psychosocial interventions, and administration of opiate treatments in a clinical setting. Psychosocial treatment included medical and psychiatric follow-up, primary health care, psychosocial interventions, and, if necessary, administration of pharmacotherapy in a clinical setting” |  |  |
|  |  | Number of participants allocated to each group: 60 in BI, 52 in TAU |  |  |
|  |  | Duration of the intervention (mean ± SD): 16 ± 4.7 minutes |  |  |
|  |  | Duration of follow-up: 3 and 9 months |  |  |
|  |  | Country of origin, setting: specialized outpatient clinic in the Division of Substance Abuse of the University Hospitals of Geneva, Switzerland |  |  |
|  |  |  |  | • Interventionist trained by studying the manual and watching MI tapes from Project MATCH |
|  |  |  | (2) MI-group: focus: alcohol, risky behaviors, MI spirit; by trained MI specialists, that is, a PhD-prepared psychologist conducted primarily the MI-group sessions. Content of the individual and group sessions was identical, guided by a detailed protocol and biweekly meetings with the investigator and therapists. The average number of participants was 6 (range 5–7) |  |
|  |  |  | (3) MI-single: focus: alcohol, risky behaviors, MI spirit; a MSW-prepared researcher conducted primarily the individual MI sessions |  |
|  |  |  | Number of participants allocated to each group: HHP: N = 87; MI group: N = 79; MI single: N = 90 |  |
|  |  |  | Duration of the intervention: 3 x 60-minute sessions, spaced 2 weeks apart |  |
|  |  |  | Duration of follow-up: 6 months |  |
|  |  |  | Country of origin, setting: 5 methadone treatment sites in California, USA |  |
|  |  |  |  |  |
|  | Route of delivery: treatments were manual-guided, 4 doctoral-level psychologists conducted CBT, 2 masters-level clinicians conducted TSF. |  |  |  |
|  |  |  |  | • Standard delivery of the MI protocol |
|  |  |  |  | • Adherence monitoring by: MI checklist completed by the therapist after each session and audiotapes of sessions were randomly reviewed by a supervisor trained in MI |
|  |  |  |  | (2) Control: assessment only, approximately 3 hours |
|  |  |  |  | Number of participants allocated to each group: 95 in MI, 92 in control group |
|  |  |  |  | Duration of the intervention: 2 therapist sessions, 1 month apart; 1st session: 60 minutes, 2nd session: 30 to 45 minutes |
|  |  |  |  | Duration of follow-up: 1 and 6 months |
|  |  |  |  | Country of origin, setting: NEP clients, study site: Rhode Island Hospital in Providence, USA |
|  |  |  |  |  |
|  | Number of participants allocated to each group: 25 in CBT plus no medication; 19 in TSF plus no medication |  |  |  |
|  | Duration of the intervention: 12 weeks, 16 individual sessions |  |  |  |
|  | Duration of follow-up: 12 weekly assessments within-treatment, and at 1, 3, 6, 12 months. |  |  |  |
|  | Country of origin, setting: a non-profit substance abuse treatment centre (APT foundation) affiliated with Yale University in New Haven, Connecticut |  |  |  |
| **Outcomes** | 1.1.1 Alcohol abstinence as maximum number of weeks of consecutive alcohol abstinence during treatment | 2.1.1 Alcohol use as AUDIT scores at 3 months | 3.1.1 Alcohol use (unpublished) as number of standard drinks consumed per day over the last 30 days | 5.1.1 Alcohol use as number of days in the past 30 days with alcohol use at 1 month |
|  |  | 2.1.2 Alcohol use as AUDIT Scores at 9 months |  |  |
|  |  |  |  | 5.1.2 Alcohol use as number of days in the past 30 days with alcohol use at 6 months |
|  |  |  | 3.1.2 Illicit drug use (unpublished) as frequency of drug use (as measured by ASI drug) |  |
|  |  |  |  | 5.2.1 Alcohol use as 25% reduction of drinking days in the past 30 days |
|  | 1.1.2 Illicit drug abstinence as maximum number of weeks of consecutive abstinence from cocaine during treatment | 2.1.3 Alcohol use as number of drinks per week at 3 months (number of glasses of alcohol per week, 1 glass: 10 g of alcohol; wine = 100 mL; beer = 250 mL; spirits = 25 mL) |  |  |
|  |  |  |  | 5.2.2 Alcohol use as 50% reduction of drinking days in the past 30 days |
|  |  |  | 3.1.3 Illicit drug use (unpublished) as a composite drug score (frequency*severity for all drugs taken) |  |
|  |  |  |  | 5.2.3 Alcohol use as 75% reduction of drinking days in the past 30 days |
|  | 1.2.1 Alcohol abstinence as number achieving 3 or more weeks of consecutive alcohol abstinence during treatment |  |  |  |
|  |  | 2.1.4 Alcohol use as number of drinks per week at 9 months |  |  |
|  |  |  | 3.2.1 Alcohol use as > 50% reduction in number of standard drinks consumed per day over the last 30 days |  |
|  |  | 2.2.1 Alcohol use as decreased alcohol use at 3 months |  |  |
|  | 1.2.2 Illicit drug abstinence as number achieving 3 or more weeks of consecutive abstinence from cocaine during treatment |  |  |  |
|  |  |  |  | 5.2.4 Alcohol use as 1 or more drinking days’ reduction in the past 30 days |
|  |  | 2.2.2 Alcohol use as decreased alcohol use at 9 months |  |  |
|  | 1.2.3 Alcohol abstinence during follow-up year |  |  | 5.2.5 Alcohol use as 7 or more drinking days’ reduction in the past 30 days |
|  |  |  | 3.2.2 Alcohol abstinence as abstinence from alcohol over the last 30 days |  |
|  |  | 2.2.3 and 2.2.4 Increased or unchanged alcohol use at 3 and 9 months (that is, reverse of the above) |  |  |
|  | 1.2.4 Illicit drug abstinence as abstinence from cocaine during follow-up year |  |  |  |
|  |  |  | Outcomes 4.1.1 to 4.2.2 refer to the individual (single) format of MI |  |
